# Uniaxial Tensile Strain Induced the Enhancement of Thermoelectric Properties in *n*-Type BiCuO*Ch* (*Ch* = Se, S): A First Principles Study

**DOI:** 10.3390/ma13071755

**Published:** 2020-04-09

**Authors:** Chunpeng Zou, Chihou Lei, Daifeng Zou, Yunya Liu

**Affiliations:** 1 Key laboratory of Low Dimensional Materials and Application Technology of Ministry of Education, School of Materials Science and Engineering, Xiangtan University, Xiangtan 411105, China; 2 Department of Aerospace and Mechanical Engineering, Saint Louis University, Saint Louis, MO 63103, USA; 3 School of Physics and Electronic Science, Hunan University of Science and Technology, Xiangtan 411201, China; 4 Shenzhen Key Laboratory of Nanobiomechanics, Shenzhen Institutes of Advanced Technology, Chinese Academy of Sciences, Shenzhen 518055, China

**Keywords:** BiCuOSe, BiCuOS, strain, thermoelectric properties, electronic structure

## Abstract

It is well known that the performance of thermoelectric measured by figure of merit *ZT* linearly depends on electrical conductivity, while it is quadratic related to the Seebeck coefficient, and the improvement of Seebeck coefficient may reduce electrical conductivity. As a promising thermoelectric material, BiCuO*Ch* (*Ch* = Se, S) possesses intrinsically low thermal conductivity, and comparing with its *p*-type counterpart, *n*-type BiCuO*Ch* has superior electrical conductivity. Thus, a strategy for increasing Seebeck coefficient while almost maintaining electrical conductivity for enhancing thermoelectric properties of *n*-type BiCuO*Ch* is highly desired. In this work, the effects of uniaxial tensile strain on the electronic structures and thermoelectric properties of *n*-type BiCuO*Ch* are examined by using first-principles calculations combined with semiclassical Boltzmann transport theory. The results indicate that the Seebeck coefficient can be enhanced under uniaxial tensile strain, and the reduction of electrical conductivity is negligible. The enhancement is attributed to the increase in the slope of total density of states and the effective mass of electron, accompanied with the conduction band near Fermi level flatter along the Γ to Z direction under strain. Comparing with the unstrained counterpart, the power factor can be improved by 54% for *n*-type BiCuOSe, and 74% for *n*-type BiCuOS under a strain of 6% at 800 K with electron concentration 3 × 10^20^ cm^−3^. Furthermore, the optimal carrier concentrations at different strains are determined. These insights point to an alternative strategy for superior thermoelectric properties.

## 1. Introduction

The development of new energy materials is on the rise in recent decades and attracts more and more attention owing to the impact on the environment [1,2]. As a newly promising material, thermoelectric material possesses a prominent advantage for being able to directly convert heat energy into electricity [3,4,5,6,7]. The performance of thermoelectric materials is quantified by a dimensionless constant known as the figure of merit *ZT = S*^2^*σT/κ*, with *S* being the Seebeck coefficient, *σ* being the electrical conductivity, *κ* being the thermal conductivity, and *T* being the absolute temperature. To achieve a higher *ZT*, it is necessary to enhance the power factor *S*^2^*σ* or reduce the thermal conductivity.

Newly discovered thermoelectric materials BiCuOSe and BiCuOS [BiCuO*Ch* (*Ch* = Se, S)] have attracted attentions due to their intrinsically low thermal conductivity [8,9,10,11], whose conductive layers (Cu_2_Se_2_)^2−^ or (Cu_2_S_2_)^2−^ are alternately stacked with an insulating layer (Bi_2_O_2_)^2+^, composing of the ZrSiCuAs structure type, and this layered structure may be an important factor affording its low thermal conductivity [12,13]. Because of the low lattice thermal conductivity, efforts to improve thermoelectric performance *ZT* of these compounds have mainly focused on enhancing their power factor *S*^2^*σ*. To obtain high power factor, several approaches to increase the electrical conductivity of BiCuO*Ch*, such as doping [14,15], pressure [16], and strain [17], have been attempted. As the electrical conductivity, *σ*, and Seebeck coefficient, *S*, are coupled, improving electrical conductivity will reduce Seebeck coefficient [1]. Compared with *p*-type BiCuOSe, *n*-type BiCuOSe possesses higher electrical conductivity [16]. On the other hand, it is also noticed that the power factor, *S*^2^*σ*, depends linearly on electrical conductivity, *σ*, but quadratically on Seebeck coefficient *S*. Thus, it is an alternative pathway to achieve the enhancement of power factor for *n*-type BiCuO*Ch* via enhancing Seebeck coefficient while keeping electrical conductivity with only slight reduction.

Recently, band engineering has proved to be an effective method to improve the electronic transport properties of thermoelectric materials [18]. Some works have verified that the transport properties can be tuned by strain, including both in-plane biaxial strains and out-of-plane uniaxial compressive strain [17,19,20]. It is also well known that materials with micro/nanopillar array structures are always subjected to out-of-plane uniaxial strain imposed by surrounding matrix [21,22], and thus applying uniaxial tensile strain to thermoelectric materials can be easily realized in pillar array structures in experiments by using appropriate matrix. As uniaxial tensile strain can also tune the electronic structures of thermoelectric materials, it may provide a possible pathway to enhance thermoelectric performance. Thus, it is necessary to explore how uniaxial tensile strain affects the electronic structures and transport properties of *n*-type BiCuO*Ch*, and it is expected that the present work will offer a useful pathway to tune the electronic structures leading to the enhancement of the thermoelectric performances of *n*-type BiCuO*Ch*. In this work, we study the effects of uniaxial tensile strain along *c* axis on the electronic structures of *n*-type BiCuO*Ch* by using first-principles calculations, and investigate the thermoelectric properties of *n*-type BiCuO*Ch* under uniaxial tensile by semiclassical Boltzmann transport theory. We find that uniaxial tensile strain can be utilized as an alternative pathway to enhance the thermoelectric properties of *n*-type BiCuO*Ch*.

## 2. Computational Details

Density function theory (DFT) has been adopted to calculate the lattice constants and electronic structures of BiCuO*Ch* (*Ch* = Se, S) under strain constraint, which are implemented in the Vienna ab initio Simulation Package (VASP) [23,24,25,26]. The projector augmented wave (PAW) method is chosen with Perdew–Burke–Ernzerhof (PBE) generalized gradient approximation (GGA) exchange-correlation potential [27]. The plane-wave basis sets with a kinetic energy cut-off of 550 eV are used in the calculations. The relaxing force is set to be 10^−3^ eV Å^−1^. The convergence energy criterion is set to be 10^−6^ eV per unit cell. The generalized gradient approximation (GGA) always underestimates the exchange-correlation effect of the strongly localized Cu 3*d* electrons. To resolve this issue, DFT + *U* is adopted to adjust the on-site Coulomb interactions [28,29], and it is an effective solution to deal with the band gaps of semiconductors with Cu [30,31]. In this work, we set *U* = 4 eV for Cu 3*d* state based on our previous investigations on BiCuOSe [13]. BiCuO*Ch* includes heavy metal element Bi, thus the spin-orbit coupling (SOC) is considered in our calculations, which takes into account of their relativistic effect.

Based on the simulated electronic structures, the thermoelectric transport properties of *n*-type BiCuO*Ch* are calculated by the semiclassical Boltzmann theory via the BoltzTraP package [32,33]. Similar to treatments employed in literature [13], constant relaxation time approximation is used in the calculations of thermoelectric transport properties, because the scattering time of most semiconductors is insensitive to energy [34]. A Monkhost–Pack mesh of 31 × 31 × 13 k-point is used to obtain the accurate thermoelectric transport properties of BiCuO*Ch* [35,36].

## 3. Results and Discussion

### 3.1. Crystal Structures

**Figure 1:**
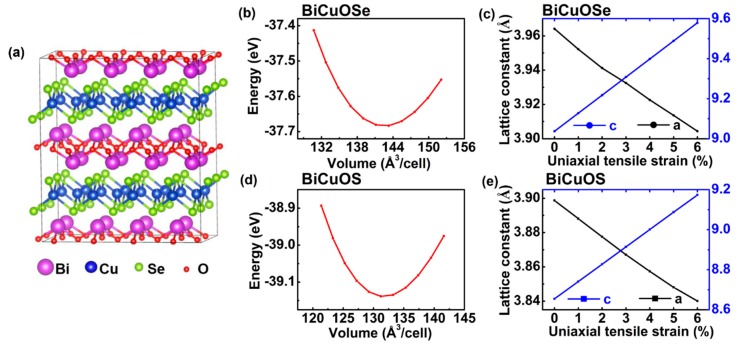
(**a**) Crystal structure of BiCuOSe. Variation of energy with respect to volume during optimizing structures of unstrained (**b**) BiCuOSe and (**d**) BiCuOS. The optimized lattice constants of (**c**) BiCuOSe and (**e**) BiCuOS as function of uniaxial tensile strain.

The typical crystal structure of BiCuOSe is shown in Figure 1a, in which the (Cu_2_Se_2_)^2−^ layers and the (Bi_2_O_2_)^2+^ layers are alternatively stacked together along the *c* axis direction. BiCuOS, whose crystal structure is similar to that BiCuOSe, is not shown here. As we can see from Figure 1a, it belongs to the layered structure, and can exhibit unique thermoelectric properties: the (Cu_2_*Ch*_2_)^2−^ layers can be considered as the conductive layers that are responsible for electrical conductivity, and the (Bi_2_O_2_)^2+^ layers can be treated as charge reservoir layers that are expected to have a large Seebeck coefficient. In addition, such layered structure also can lead to a low thermal conductivity [16,32]. In this work, the effects of uniaxial strain on the electronic structures, and thus the thermoelectric properties of *n*-type BiCuO*Ch*, are studied, with the uniaxial strain imposed along the c axis defined by $\Delta c=(c-c_{0})/c_{0}$. Notice that *c* and $c_{0}$ are the optimized lattice constants for BiCuO*Ch* under strained and unstrained states.

The optimized lattice constants are determined according to energy minimization. For example, the variations of energy with respect to volume for unstrained BiCuO*Ch* (*Ch* = Se, S) are plotted in Figure 1b,d, where the optimized lattice constants with energy minimization are *a* = *b* = 3.9641 Å and *c* = 9.0371 Å for unstrained BiCuOSe, and *a* = *b* = 3.8987 Å and *c* = 8.6546 Å for unstrained BiCuOS, as tabulated in Table 1. These theoretical calculations agree well with the corresponding experimentally measured values [37] listed in Table 1. Notice that BiCuOSe has larger lattice constants than BiCuOS due to the larger atomic radius of Se. The optimized lattice constants under different uniaxial tensile strains are plotted in Figure 1c,e. It is observed that an increase in uniaxial tensile strain leads to an increase in the out-of-plane lattice constant *c* of BiCuOSe but a decrease in the in-plane lattice constant *a*. Similar trends with respect to uniaxial tensile strain can also be observed for the lattice constants of BiCuOS. Thus, the parameters of the crystal structures of both BiCuOSe and BiCuOS can be tuned via uniaxial tensile strain, which may affect the electronic structures and the thermoelectric properties.

### 3.2. Electronic Structures

**Figure 2:**
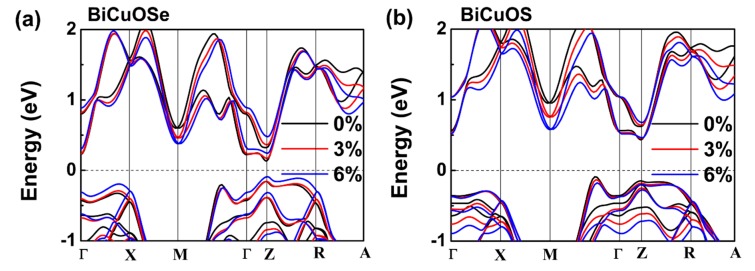
Band structures of (**a**) BiCuOSe and (**b**) BiCuOS at different uniaxial tensile strains.

The characteristics of the band structure are relevant to the thermoelectric transport properties. To gain insight into the band structures tuned by uniaxial tensile strain, the band structures of BiCuOSe and BiCuOS with and without strain are plotted along several high symmetry points in the Brillion zone in Figure 2. The calculation results indicate that both the conduction band minimum and the valence band maximum are located at the Z point, meaning that BiCuOSe has a direct band gap. BiCuOS shows an indirect band gap, whose conduction band minimum is located at the Z point, but whose valence band maximum is located between the M point and Γ point. Of particular interest to us is *n*-type BiCuO*Ch*, whose thermoelectric transport properties rely on conduction bands of band structures, allowing us to focus on the changes in conduction bands under uniaxial tensile strain. In Figure 2a, as the uniaxial tensile strain is increasing, the conduction band near Fermi level of BiCuOSe becomes flatter from Γ to Z direction, and the energy at M point is significantly decreased. It has been reported that variations in the conduction band may affect the transport properties of *n*-type BiCuOSe [38]. The changes in band structure of BiCuOS under tensile strain are similar to those of BiCuOSe under tensile strain. Note that the energy variations of conduction band at M point and Z point under strain have significant differences. The energy decreases more obviously at M point, whereas the energy increases mildly at Z point. Considering thermoelectric transport properties closely related to band structures, those changes in the conduction band near the Fermi level may influence the thermoelectric properties of *n*-type BiCuOSe and BiCuOS.

**Figure 3:**
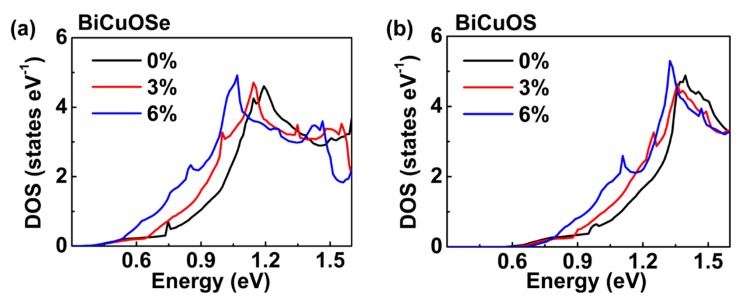
Total density of states (DOS) of (**a**) BiCuOSe and (**b**) BiCuOS under different uniaxial tensile strains.

In Figure 3, the density of states (DOS) of both BiCuOSe and BiCuOS under different uniaxial tensile strains are plotted, and it is observed that the DOS curves in conduction bands close to the Fermi level have steeper slopes when uniaxial tensile strain increases, which is consistent with the energy variations around conduction band minimum. Larger slope of DOS near Fermi level is beneficial for promoting Seebeck coefficient [16], suggesting that the Seebeck coefficients of BiCuSeO and BiCuOS may be enhanced under uniaxial tensile strain.

**Figure 4:**
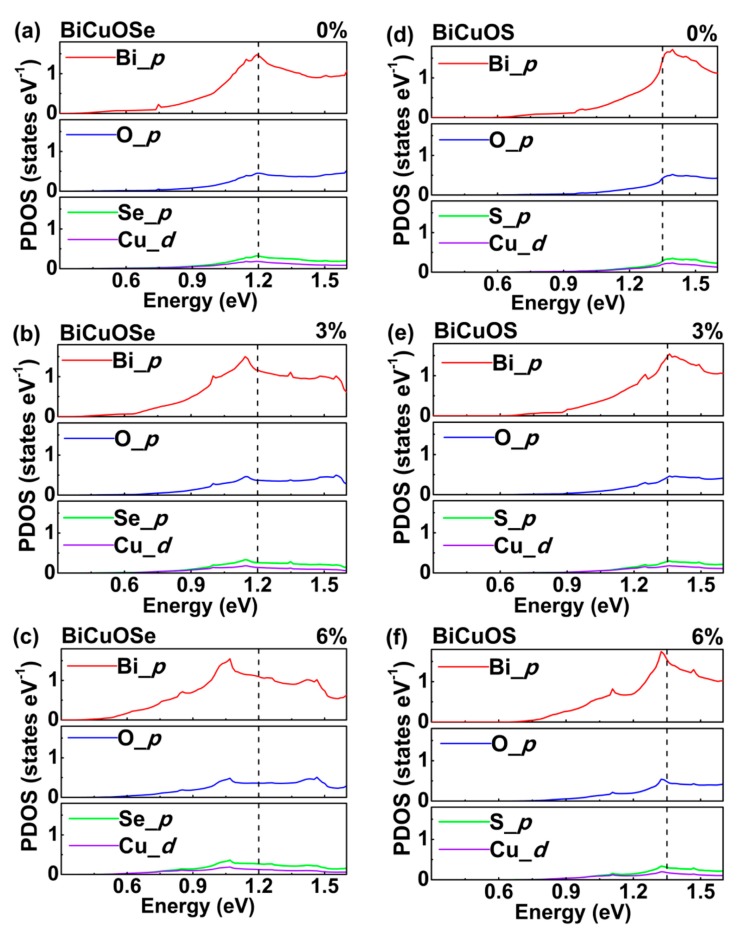
Projected density of states (PDOS) of (**a**–**c**) BiCuOSe and (**d**–**f**) BiCuOS subjected to different uniaxial tensile strains: (**a**,**d**) 0%, (**b**,**e**) 3%, and (**c**,**f**) 6%. The dash lines located at 1.2 eV for BiCuOSe and 1.35 eV for BiCuOS marked as guides for distinguishing the shifts of PDOS.

To examine the impact of uniaxial tensile strain on the DOS in detail, the projected density of states (PDOS) are calculated in Figure 4 to show the density of states in different orbitals for each atom. In both BiCuOSe and BiCuOS, Figure 4a,d indicates that the *p* orbital of Bi atoms contributes to the major part of the DOS around the conduction band minimums near Fermi level, whereas the contributions of O atoms, Se atoms, and Cu atoms are relatively small. In Figure 4b, when BiCuOSe is imposed by uniaxial tensile strain, PDOS of each atom is shifted to lower energy, where a dashed line at 1.2 eV is marked as a guide for distinguishing the shifts. For the case of BiCuOS, PDOS is also shifted to lower energy with the increase in the uniaxial tensile strain.

**Figure 5:**
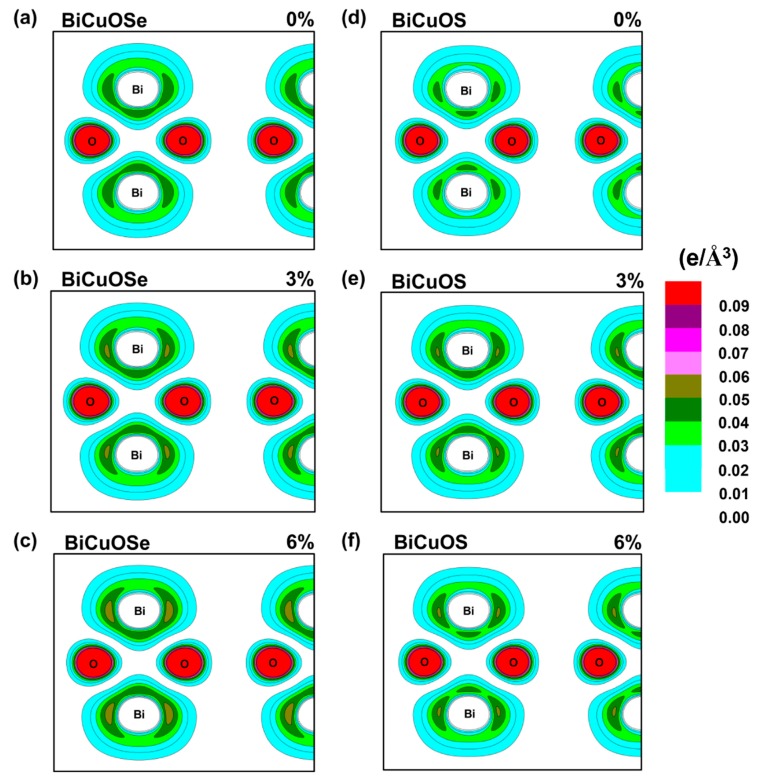
Contour plots of partial charge density of the conduction bands near Fermi level (0–2 eV) on the Bi-O-Bi plane subjected to different strains. (**a**–**c**) BiCuOSe and (**d**–**f**) BiCuOS. (**a**,**d**) 0%, (**b**,**e**) 3%, and (**c**,**f**) 6%.

To further illustrate the influences of uniaxial tensile strain on the electrical conductivity of *n*-type BiCuO*Ch*, the partial charge density near Fermi level of BiCuOSe and BiCuOS under different strain states are calculated and shown in Figure 5. The partial charge density distribution is usually used to explore the nature of electrical conductivity as reported in the references [39,40]. As the electronic transport properties of *n*-type BiCuO*Ch* are determined by the conduction bands near Fermi level, only the distributions of charge of Bi atoms and O atoms of conduction bands near Fermi level (0 to 2 eV) are shown. From the partial charge density without strain in Figure 5a, we can see the obvious antibonding characteristics between Bi and Bi atoms due to the lack of charge density between them, which determines the electrical conductivity of *n*-type BiCuO*Ch*, consistent with previous reports [16]. As the strain increases from 0 to 6%, as shown in Figure 5a–c, the charge densities around the Bi atoms slightly increases, suggesting that the antibonding of Bi–Bi becomes weakened slightly under strain, which can lead to a slight decreasing trend in electrical conductivity of *n*-type BiCuOSe under increasing uniaxial tensile strain. The trends of changes in partial charge density in *n*-type BiCuOS under strains are shown in Figure 5d–f. The scenarios for BiCuOS are similar and we will not discuss them in detail.

### 3.3. Thermoelectric Properties

**Figure 6:**
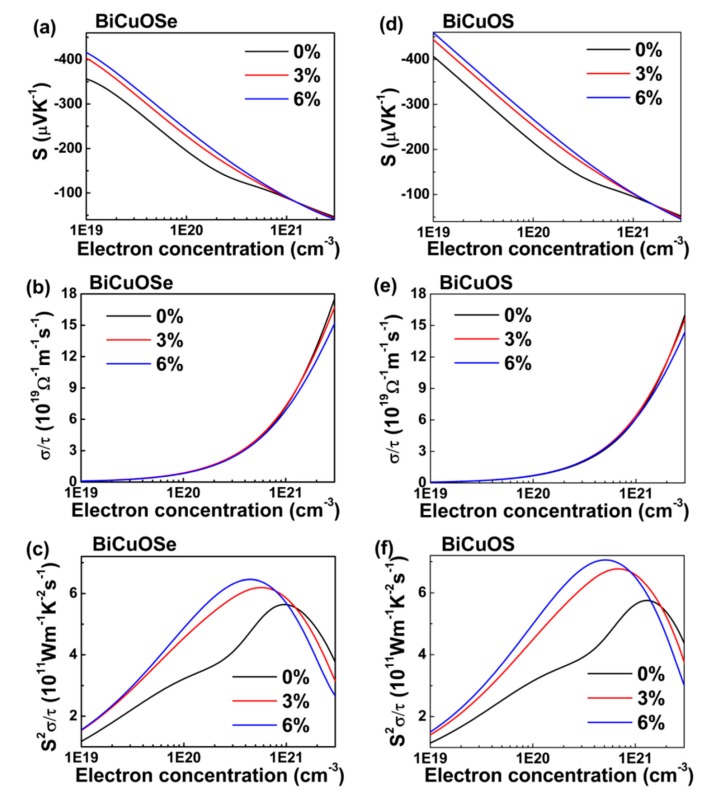
Thermoelectric properties of *n*-type (**a**–**c**) BiCuOSe and (**d**–**f**) BiCuOS as a function of electron concentration subject to different uniaxial tensile strains at 800 K. (**a**,**d**) Seebeck coefficient *S*, (**b**,**e**) electrical conductivity with respect to relaxation time *σ*/*τ*, and (**c**,**f**) power factors with respect to relaxation time *S*^2^*σ*/*τ*.

Thermoelectric properties are correlated with electronic structures. After examining the effects of uniaxial tensile strain on the electronic structures of BiCuO*Ch*, we further investigate the effects of uniaxial tensile strain on the thermoelectric properties, which are estimated by solving the Boltzmann transport equation. Notice that the relaxation time *τ* cannot be determined via the Boltzmann theory. Similar to previous work [32], the electrical conductivity derived with respect to the relaxation time is obtained under the assumption of constant relaxation time. The thermoelectric properties of *n*-type BiCuOSe and BiCuOS as a function of electron concentration under different uniaxial tensile strains are shown in Figure 6, where the temperature is chosen at 800 K as BiCuOSe and BiCuOS belong to medium temperature thermoelectric materials [17,37].

For the unstrained state, both *n*-type BiCuOSe and BiCuOS have negative Seebeck coefficients, and the absolute values of the Seebeck coefficients decrease with the increase in electron concentration (Figure 6a,d), whereas the electrical conductivities increase (Figure 6b,e), as expected. Opposite trends in the variations of Seebeck coefficients and electrical conductivities with respect to electron concentration result in a peak with maximum value in power factor, as shown in Figure 6c,f. Comparing with *n*-type BiCuOS, *n*-type BiCuOSe possesses a lower Seebeck coefficient but a higher electrical conductivity. It is well known that the electronic conductivity of *n*-type thermoelectric materials is mainly dominated by the conduction band near Fermi level. Combining with the DOS curves in the conduction bands close to Fermi level shown in Figure 3, the slope of DOS curves of BiCuOS is steeper than that of BiCuOSe, suggesting that *n*-type BiCuOS has higher Seebeck coefficient than *n*-type BiCuOSe, whereas the trend in electrical conductivity is just opposite [39].

In Figure 6a,d, when uniaxial tensile strain is applied to *n*-type BiCuOSe and BiCuOS, an obvious enhancement of Seebeck coefficient can be observed over a wide range of concentration, except possibly at high concentration. Quite encouragingly, the decrease of electrical conductivity is negligible, as shown Figure 6b,e. Thus, uniaxial tensile strain can be utilized to promote Seebeck coefficient while almost maintaining electrical conductivity, which significantly enhances the power factors of *n*-type BiCuOSe and BiCuOS, as evidenced in Figure 6c,f. To understand the enhancement of Seebeck coefficient under strain, we resort to changes of electronic structures under uniaxial tensile strain. According to the results of Figure 2 described above, the conduction band near Fermi level of BiCuO*Ch* becomes flatter along the Γ to Z direction under strain, suggesting that the effective mass of electron increases when strain is imposed. It is noticed that Seebeck coefficient is correlated to the effective mass according to $S=\frac{8\pi^{2}k_{B}^{2}}{3eh^{2}}m^{*}T{\left(\frac{\pi }{3n}\right)}^{2/3}$ [4], where m* is the carrier effective mass, and *k*_B_, *e*, *h*, and *n* are the Boltzmann constant, charge per electron, the Planck constant, and carrier concentration, respectively, suggesting that large effective mass results in high Seebeck coefficient. Thus, an increase in effective mass of electron under uniaxial tensile strain induces an enhancement in Seebeck coefficient. It is also noticed that the slope of DOS near Fermi level becomes steeper under uniaxial tensile strain (Figure 3), which is beneficial for increasing Seebeck coefficient [16], again implying that uniaxial tensile strain can enhance the Seebeck coefficients of *n*-type BiCuOSe and BiCuOS.

**Figure 7:**
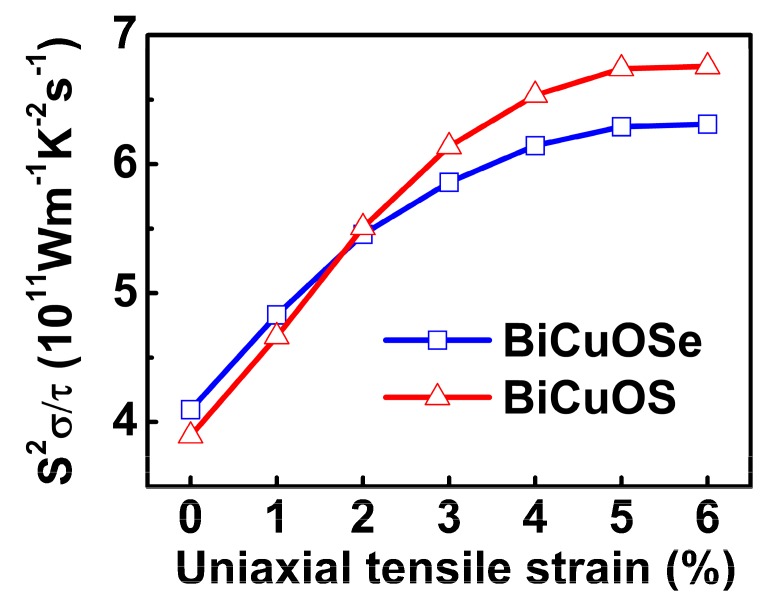
Power factors with respect to relaxation time *S*^2^*σ*/*τ* of *n*-type BiCuOSe and BiCuOS as a function of uniaxial tensile strain at 800 K with electron concentration being 3 × 10^20^ cm^−3^.

**Figure 8:**
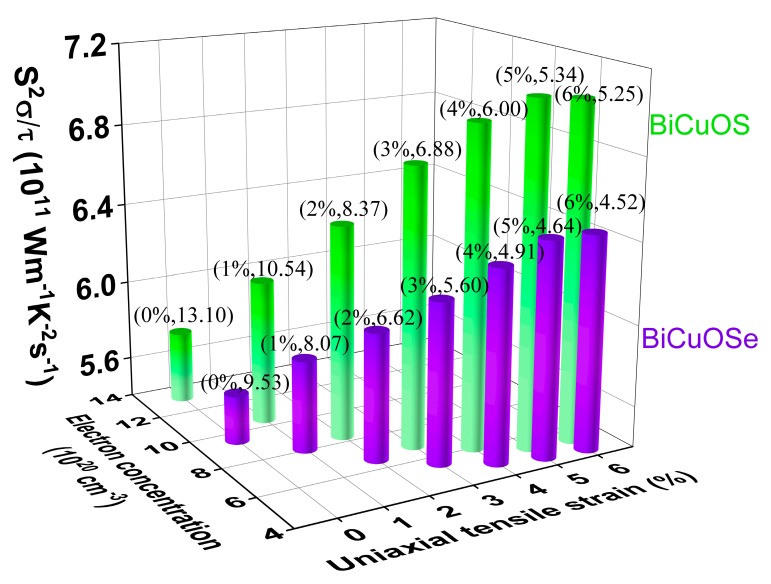
Power factors of *n*-type BiCuOSe and BiCuOS versus the optimal electron concentration for different uniaxial tensile strains at 800 K. Note that the data of (strain, optimal electron concentration) are marked on the pillars.

To exhibit the effects of uniaxial tensile strain on the thermoelectric properties more clearly, the power factors of *n*-type BiCuOSe and BiCuOS as functions of uniaxial tensile strain at fixed electron concentration are shown in Figure 7. It is seen that the power factor increases rapidly when the uniaxial tensile strain increases initially, before it reaches a plateau after a uniaxial tensile strain of approximately 5% for both *n*-type BiCuOSe and BiCuOS. Comparing with the unstrained counterpart, the power factor of *n*-type BiCuOSe is enhanced by 54% at a uniaxial tensile strain of 6%, whereas an enhancement of 74% is reached for the *n*-type BiCuOS under a uniaxial tensile strain of 6%. Furthermore, according to the curve of power factor plotted in Figure 6c,e, the optimal electron concentration, located where the power factor shows a peak value, can be determined for each uniaxial tensile strain, as shown in Figure 8. It can be seen that the *n*-type BiCuOS has a higher power factor than that of the *n*-type BiCuOSe, as the *n*-type BiCuOS possesses a higher Seebeck coefficient (Figure 6a,d). In Figure 8, it can be observed that the power factors of both *n*-type BiCuOSe and BiCuOS can be enhanced by uniaxial tensile strain, however they have different optimal electron concentrations, suggesting that uniaxial tensile strain is an alternative pathway to induce superior thermoelectric properties.

## 4. Conclusions

In summary, the electronic structures of BiCuO*Ch* under uniaxial tensile strain have been investigated based on first-principles calculations, and the strain-dependent thermoelectric properties of *n*-type BiCuO*Ch* were then estimated by semiclassical Boltzmann transport theory. The electrical transport property results show that the Seebeck coefficient can be increased under uniaxial tensile strain, while the decrease of electrical conductivity is negligible. The calculations of electronic structures indicate that the conduction band near Fermi level becomes flatter along Γ to Z direction under strain, leading to an increase in the slope of the total density of states and the effective mass of electron, resulting in the enhancement of Seebeck coefficient. Comparing with its unstrained counterpart, the power factor is improved by 54% for *n*-type BiCuOSe and 74% for *n*-type BiCuOS under a strain of 6% at 800 K with electron concentration 3 × 10^20^ cm^−3^. The optimal carrier concentrations at different strains have been also determined. These insights offer an alternative strategy for enhancing the thermoelectric properties of *n*-type BiCuO*Ch*.

## Figures and Tables

**Table 1:** Comparison of lattice constants of unstrained BiCuO*Ch* (*Ch* = Se, S) between calculations and experiments.

		Our Work	Experiment [37]
BiCuOSe	*a* (Å) *c* (Å)	3.9641 9.0371	3.9287 8.9291
BiCuOS	*a* (Å) c (Å)	3.8987 8.6546	3.8691 8.5602
